# Supplementary material for: Alternative splicing controls teneurin-latrophilin interaction and synapse specificity by a shape-shifting mechanism
Source: Nat Commun. 2020 May 1;11:2140. doi: 10.1038/s41467-020-16029-7 (PMC7195488; doi:10.1038/s41467-020-16029-7)
Supplement: Supplementary file 1 — Supplementary Information [file 41467_2020_16029_MOESM1_ESM.pdf]

Alternative splicing controls teneurin-latrophilin interaction and synapse specificity by a shape-shifting mechanism

Jingxian Li[1,2#], Yuan Xie[1#], Shaleeka Cornelius[3,4], Xian Jiang[3,4], Richard Sando[3,4], Szymon P. Kordon[1,2], Man Pan[1], Katherine Leon[1,2], Thomas C. Südhof[3,4], Minglei Zhao[1*], Demet Araç[1,2*]

[1]Department of Biochemistry and Molecular Biology, The University of Chicago, Chicago, IL, 60637, USA.

[2]Grossman Institute for Neuroscience, Quantitative Biology and Human Behavior, The University of Chicago, Chicago, IL, 60637, USA

[3]Department of Molecular and Cellular Physiology, Stanford University, Stanford, CA, 94305, USA.

[4]Howard Hughes Medical Institute

[#]These authors have contributed equally

[*]Corresponding authors:

Minglei Zhao: mlzhao@uchicago.edu

Demet Araç: arac@uchicago.edu

Supplementary Material:

Supplementary Figures 1-7

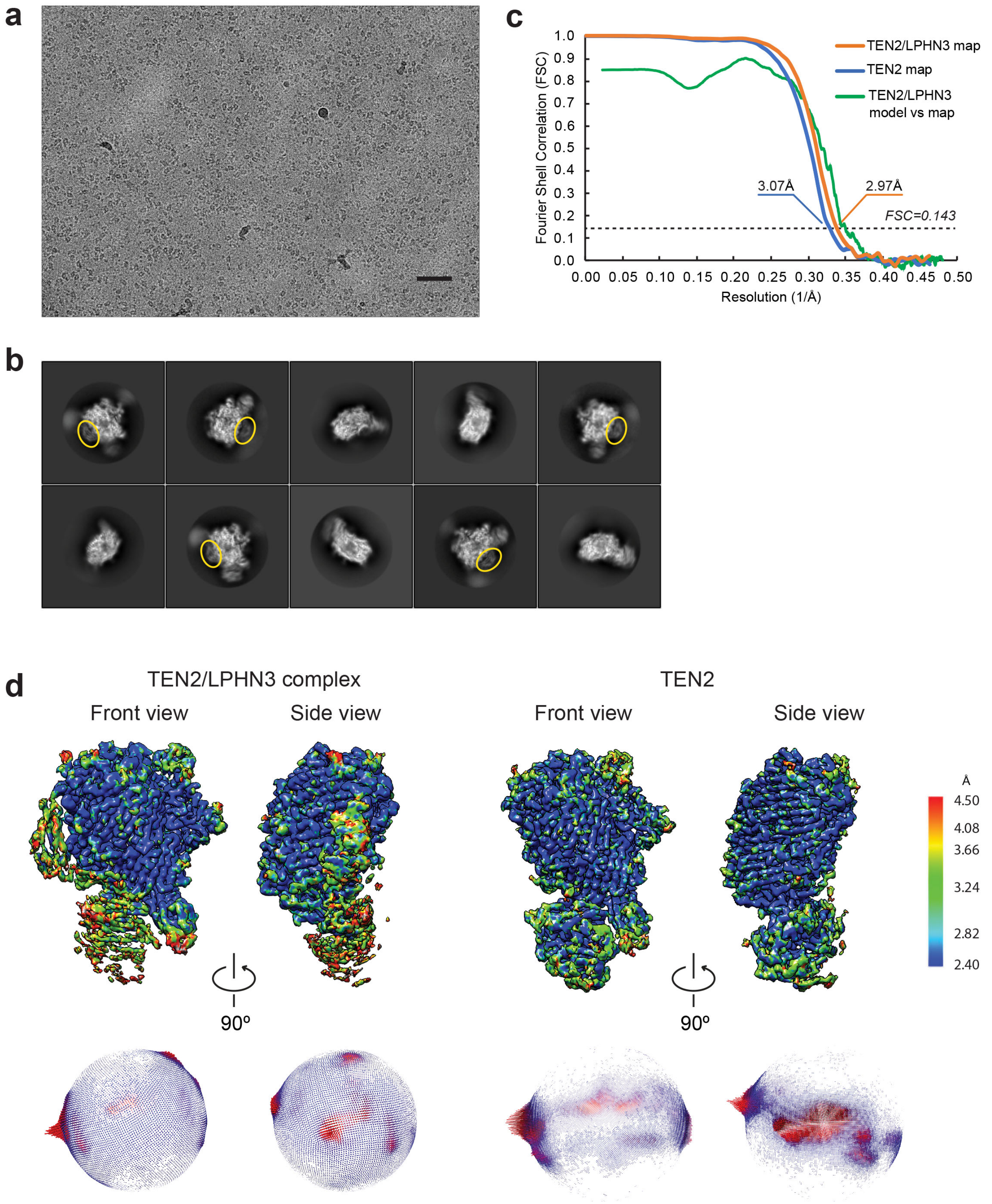

Supplementary Figure 1. Map Quality and Local Resolution. (a) A representative electron micrograph of TEN2/LPHN3 complex collected using a K3 direct detection camera (Gatan, Inc., Pleasanton, CA). Scale bar is 50 nM. (b) Representative 2D class averages of TEN2/LPHN3 complex. The diameter of the circular mask is 180 Å. The Lec domain is highlighted in yellow circles. (c) Fourier shell correlation (FSC) curves of the cryo-EM maps. The resolutions are determined using FSC=0.143 criterion after RELION post-processing. Orange: FSC curve of TEN2/LPHN3; Blue: FSC curve of TEN2; Green: Model vs. TEN2/LPHN3 map calculated using PHENIX. (d) Two views of the final 3D density maps of TEN2/LPHN3 complex and TEN2 focusing on domain 3 (propeller) are colored based on the local resolution determined by ResMap[1]. Corresponding angular distribution plots for all particles in the final maps are shown under each view.

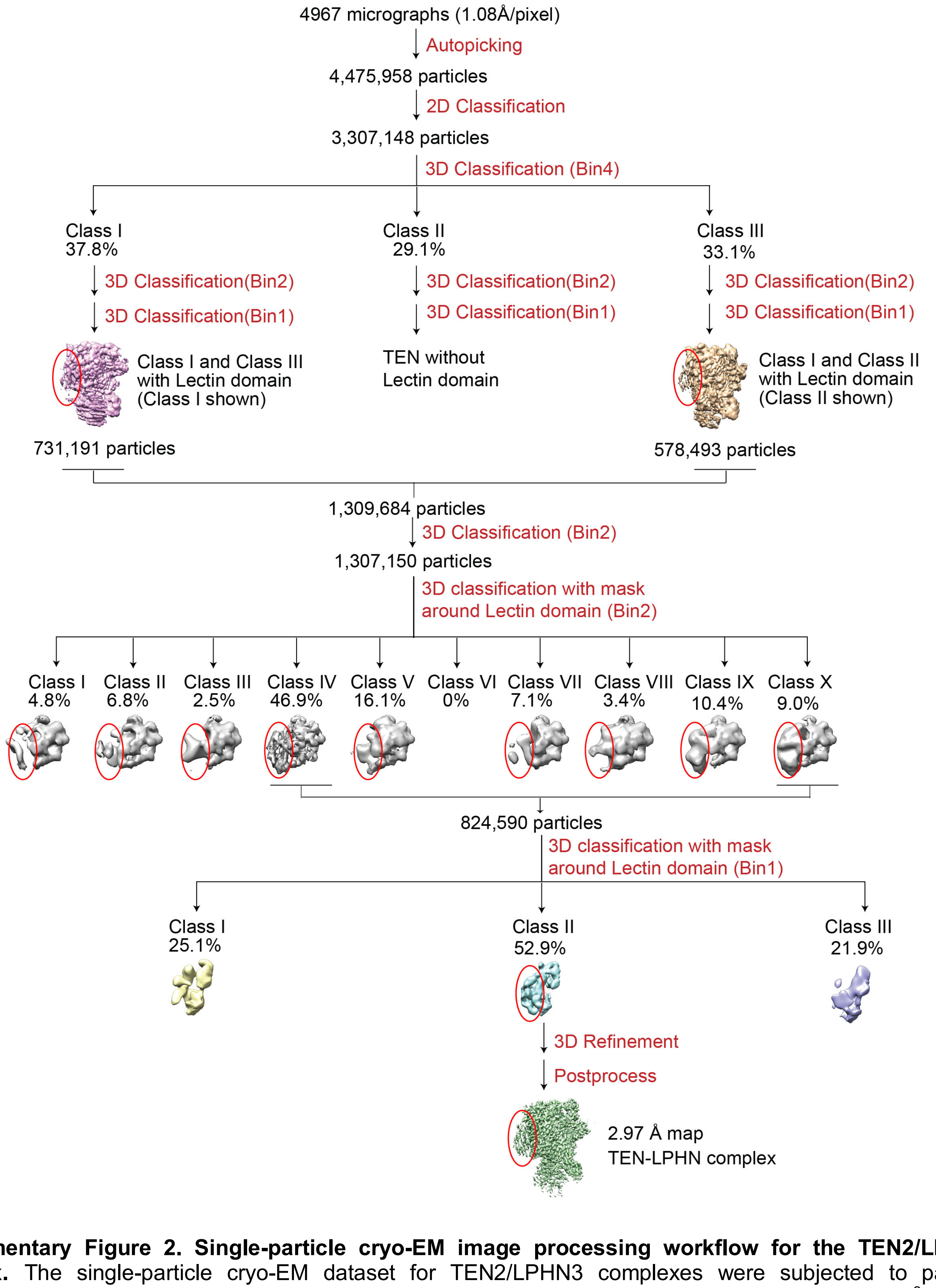

Supplementary Figure 2. Single-particle cryo-EM image processing workflow for the TEN2/LPHN3 complex. The single-particle cryo-EM dataset for TEN2/LPHN3 complexes were subjected to particle selection, 2D classification, and rounds of 3D classification using a low-pass filtered model of TEN2[2] as the initial model. Classes with clear densities of the Lec domain (highlighted in red circles) were further selected for focused 3D classification with a mask surrounding the Lec domain. The best class was selected for 3D refinement and post-processing, resulting in the final map of TEN2/LPHN3 complex at a nominal resolution of 2.97Å as determined by FSC=0.143 criterion (Supplementary Figure 1). Details are provided in the methods section.

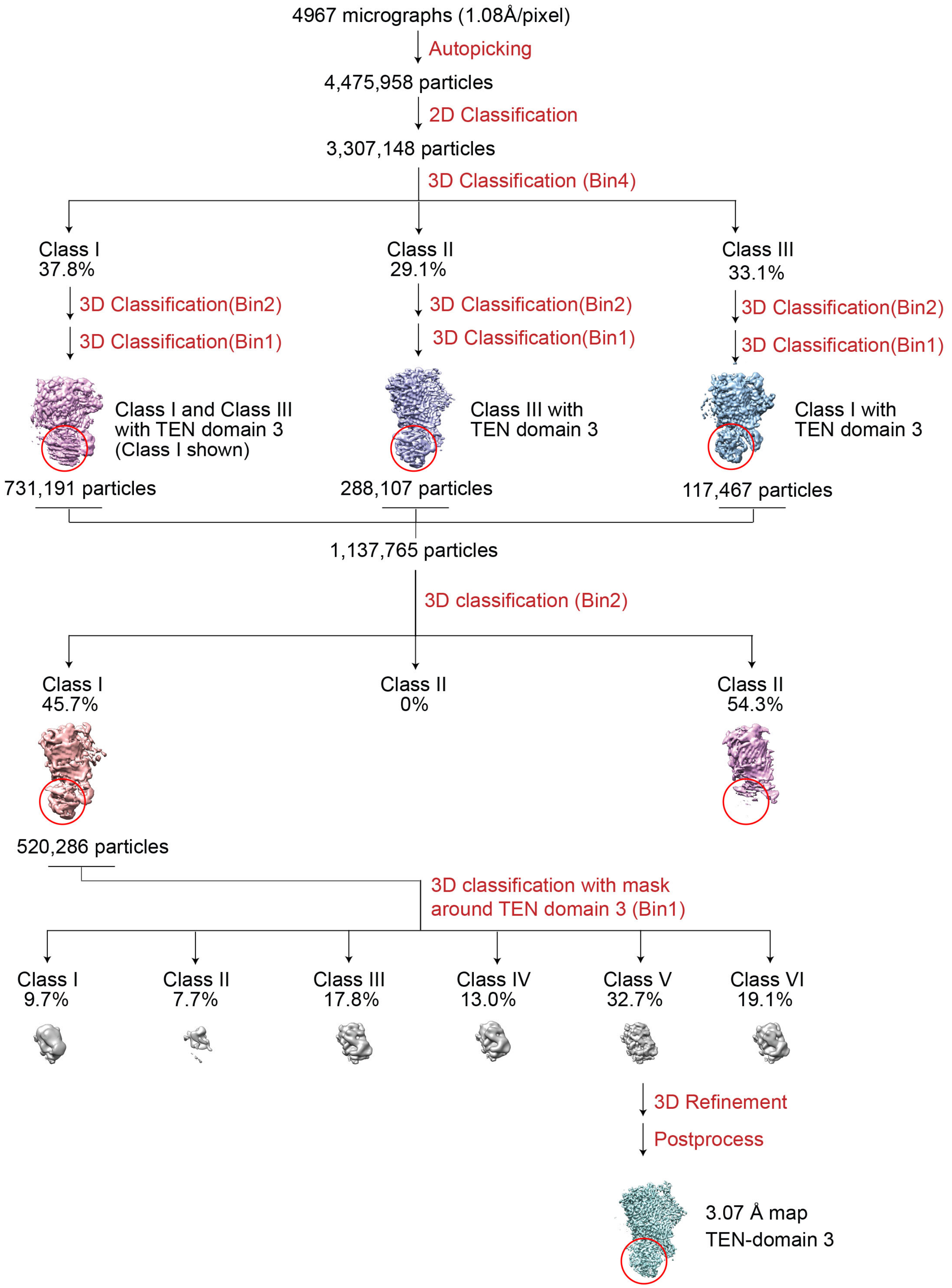

Supplementary Figure 3. Single-particle cryo-EM image processing workflow for the TEN2 ECR focusing on TEN domain 3 (propeller). After initial rounds of 3D classification using a low-pass filtered model of TEN2[2] as the initial model (Supplementary Figure 2), classes with clear densities of TEN2 domain 3 (highlighted in red circles) were further selected for a focused 3D classification with a mask surrounding domain 3. The best class was selected for 3D refinement and post-processing, resulting in a final map of TEN2 with well-resolved domain 3 at a nominal resolution of 3.07Å as determined by FSC=0.143 criteria (Supplementary Figure 1). Details are provided in the methods section.

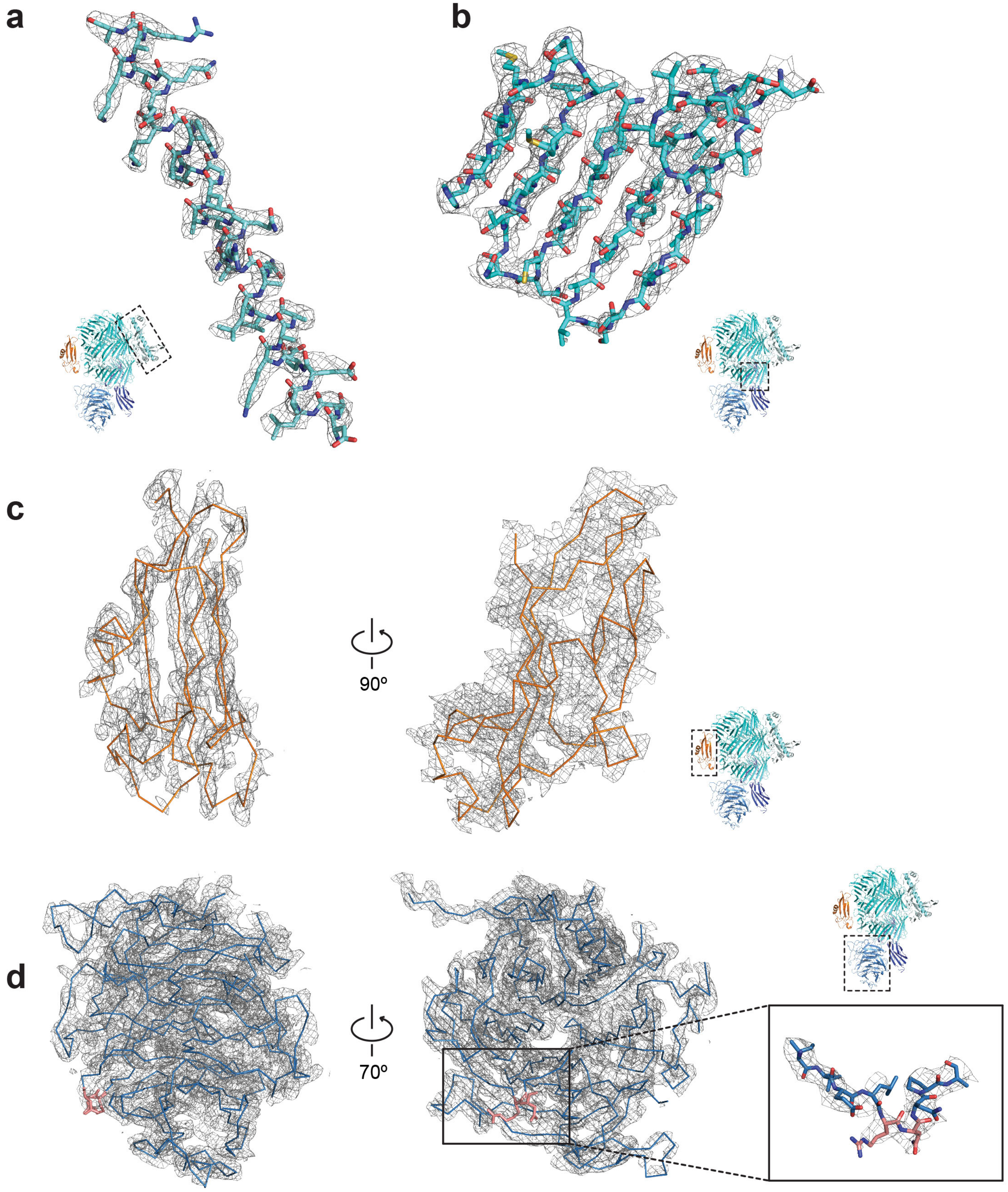

Supplementary Figure 4. Model quality. Snapshots of map versus model agreement. Each region is highlighted by a dashed box including the representative regions of an α-helix in TEN2 Tox-like domain (a), a β-sheet in TEN2 β-barrel domain (b), the Lec domain of LPHN3 (c), and the entire TEN2 β-propeller domain (d). The alternative splice site in β-propeller domain is located between Arginine 1156 and Asparagine 1157 (highlighted in pink). Contour level for individual snapshots: A, 5.0 rmsd; B, 5.0 rmsd; C, 4.0 rmsd; D, 5.0 rmsd.

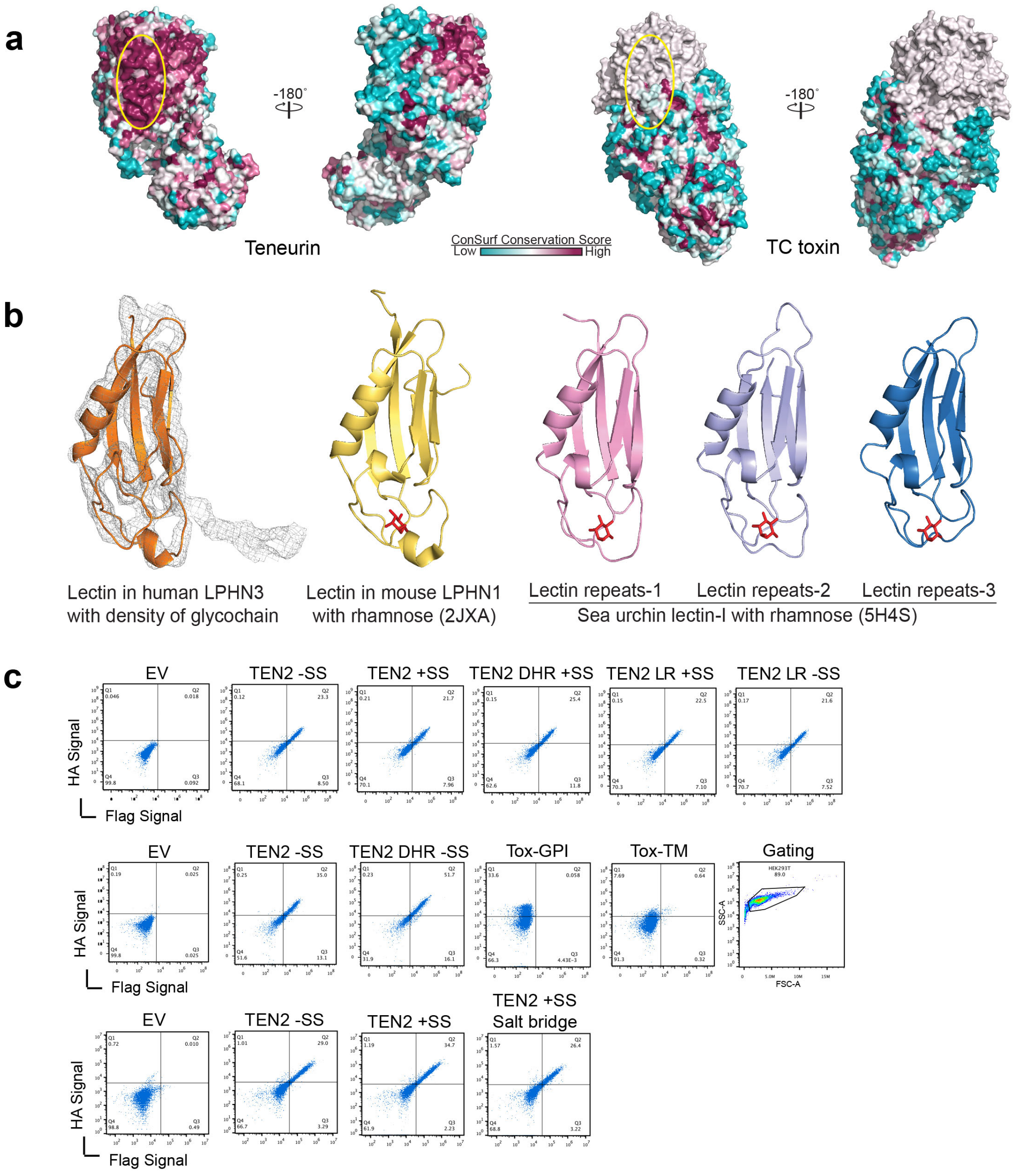

Supplementary Figure 5. Conservation, glycosylation and mutagenesis of the LPHN-binding site on TEN2. (a) Conservation analysis of TEN2 shows a highly conserved patch on the barrel surface (indicated by yellow circles) opposite to the Tox-like domain. This conserved patch is not present in bacterial toxins, implying the evolutionary importance of this region in TEN2, which may be employed to interact with partner proteins. (b) Comparison of potential sugar binding site of LPHN3 Lec domain with other SUEL-related Lec family members. Lec domain in human LPHN3 with density of glycan from human TEN2 shown in grey mesh is colored as orange; Lec domain in mouse LPHN1 from NMR model (PDB: 2JXA) with rhamnose highlighted in red is colored as yellow[3]. Three Lec repeats in sea urchin LPHN3 (PDB: 5H4S) with rhamnose highlighted in red are colored as pink, palecyan and blue, respectively[4]. All the Lec domains are aligned. Note that the glycan density from TEN2 is close to the sugar binding pocket of other Lec domains. (c) Flow cytometry analysis of cell-surface expression for full length TEN2 constructs on non-permeabilized HEK293T mammalian cells, compared to empty vector-transfected cells (EV) and cells that were transfected with mutant TEN2 constructs. Representative gating strategy to sort HEK293T cells for cultures presented in all figures related to flowcytometry experiments was shown, based on forward scatter (FSC) and side scatter (SSC). Cell populations were gated to separate cells from debris.

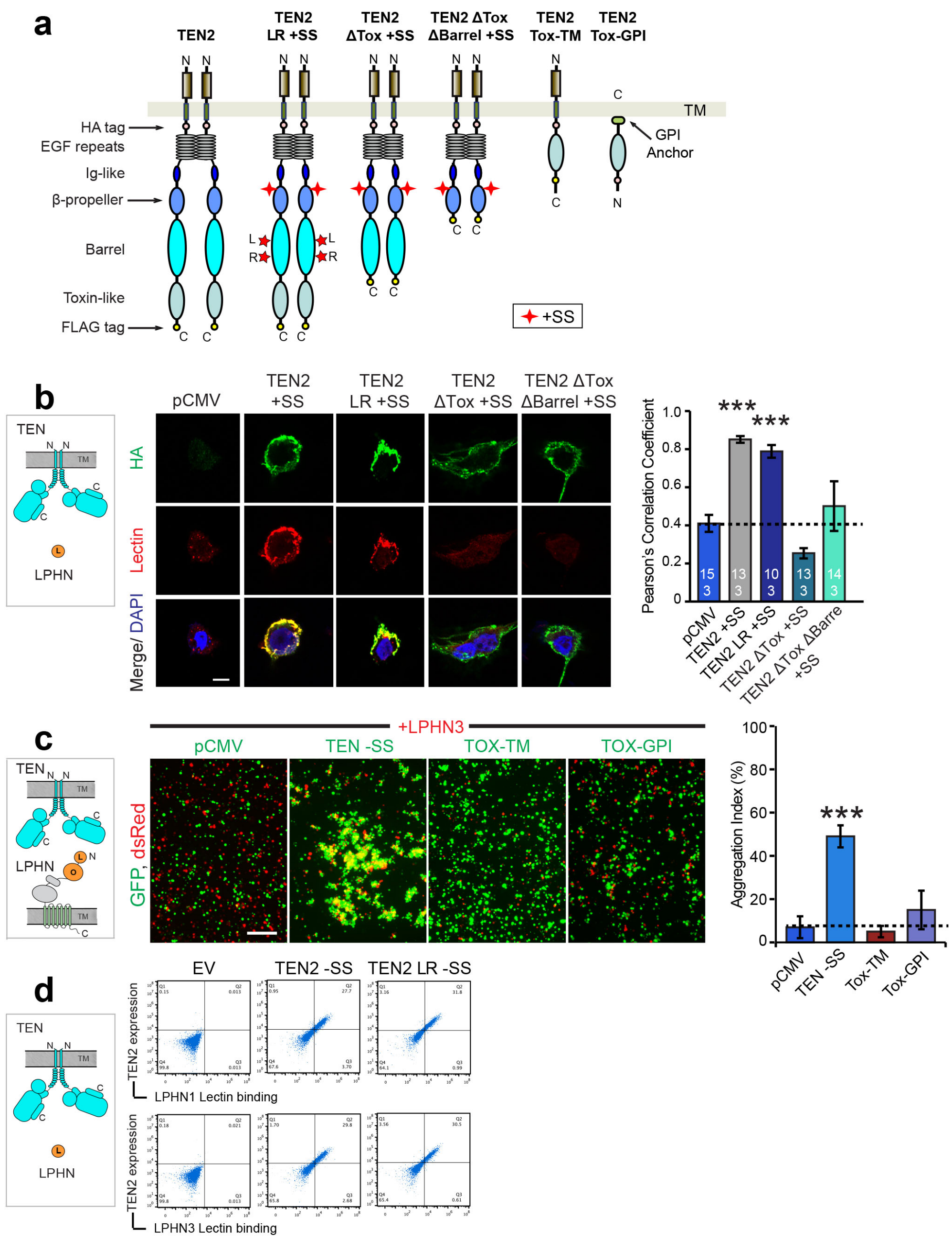

Supplementary Figure 6. LR mutation on TEN2 has no influence on LPHN3 binding; Toxin-like domain deletion and Toxin-like domain alone results. (a) Diagram for WT TEN2 +SS, Tenm2 LR +SS (L1990N, R1992T), TEN2 +SS Tox domain deletion (ΔTox), TEN2 +SS barrel and Tox domain deletion (ΔTox ΔBarrel), Toxin domain anchored to the membrane by the TM of TEN2 (Tox-TM) and Toxin domain anchored to the membrane by GPI anchor (Tox-GPI) constructs that were used in this and our previous study[2]. TEN2 LR +SS construct is generated as a positive control and it carries two point mutations in the TEN2 barrel which are not at the LPHN-binding site. The TEN2 +SS ΔTox and TEN2 +SS ΔTox ΔBarrel are the same constructs as was used in our previous study[2]. (b) Cell surface staining assays suggest that the "LR" mutations on TEN2 β-barrel has no influence on the binding between TEN2 and LPHN3. Cell surface staining assays suggest that the toxin-domain deletion abolishes LPHN binding consistent with what we published in our previous manuscript[2] Thus, we think that the TEN2 ΔTox mutant is indeed misfolded but manages to escape the protein quality control system of the cells and traffic to the cell surface. (c) Representative images for cell-aggregation assays of TEN2 -SS, Tox-TM or Tox-GPI with full-length LPHN3. TEN2 -SS induces cell aggregation with LPHN3, while Tox-TM or Tox-GPI does not. Scale bar indicates 100 μm. Quantification of cell surface binding assays are shown next to the images. (d) Wild-type (WT) and mutant TEN2 constructs were tested for surface expression in HEK293T cells as well as their ability to bind soluble LPHN1/LPHN3 Lec domain using flow cytometry. Source data are provided as a Source Data file.

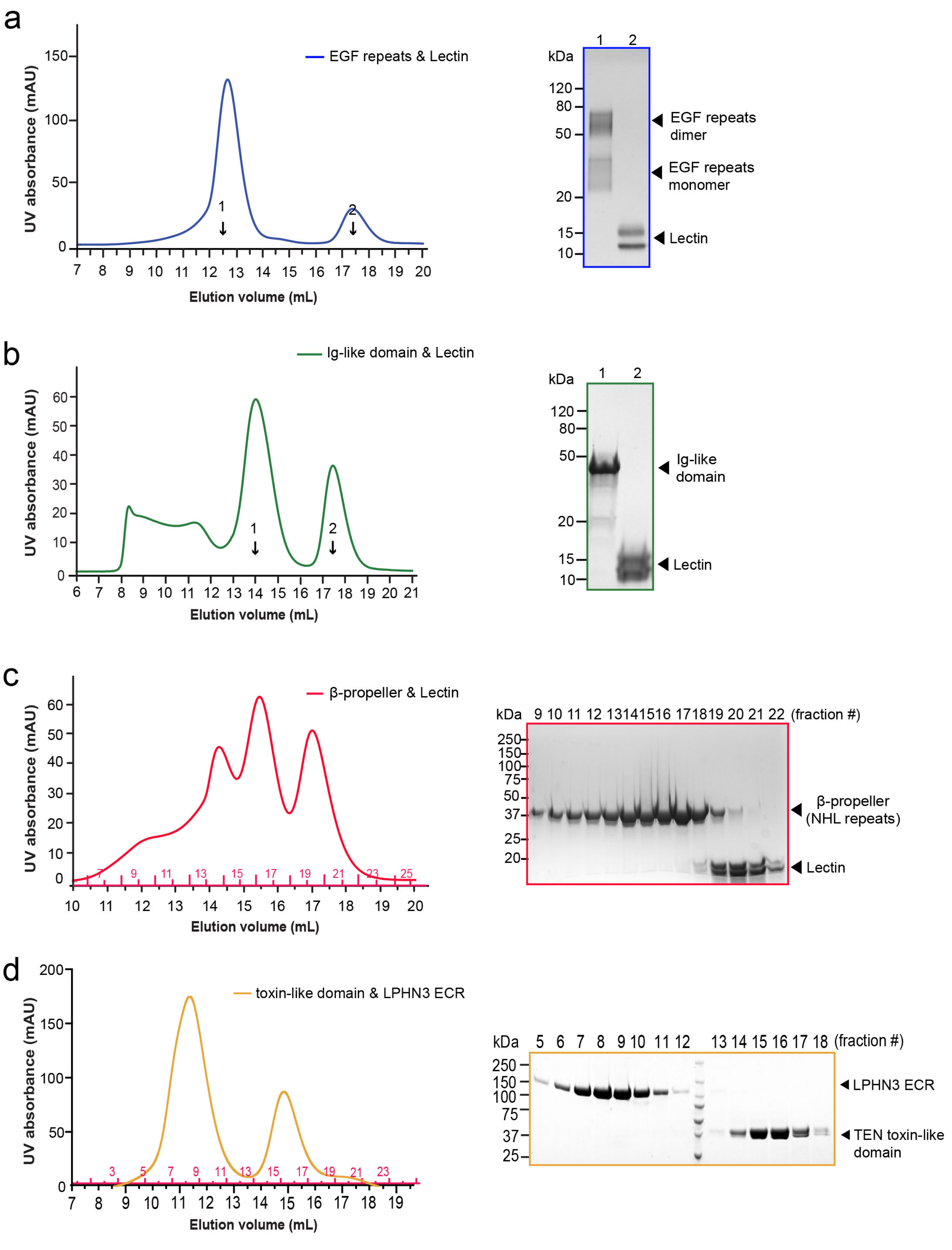

Supplementary Figure 7: LPHN does not bind to TEN2 EGF repeats, Ig-like domain, β-propeller domain or Tox-like domain. Gel filtration chromatogram and SDS-PAGE analysis of the corresponding peak fractions for the mixtures of purified LPHN3 lectin domain with purified TEN2 EGF repeats (a), TEN2 Ig-like domain (b), TEN2 β-propeller domain (c) and the corresponding peak fractions of the co-expressed LPHN3 ECR with TEN2 Tox-like domain (d). The lack of co-elution of lectin domain/ECR with the TEN2 fragments at high protein concentrations indicates no complex formation. Source data are provided as a Source Data file.

Supplementary References

1. Kucukelbir A, Sigworth FJ, Tagare HD. Quantifying the local resolution of cryo-EM density maps. *Nat Methods* **11**, 63-65 (2014).

2. Li J*, et al.* Structural Basis for Teneurin Function in Circuit-Wiring: A Toxin Motif at the Synapse. *Cell* **173**, 735-748 e715 (2018).

3. Vakonakis I, Langenhan T, Promel S, Russ A, Campbell ID. Solution structure and sugar-binding mechanism of mouse latrophilin-1 RBL: a 7TM receptor-attached lectin-like domain. *Structure* **16**, 944-953 (2008).

4. Hatakeyama T*, et al.* Carbohydrate recognition by the rhamnose-binding lectin SUL-I with a novel three-domain structure isolated from the venom of globiferous pedicellariae of the flower sea urchin Toxopneustes pileolus. *Protein Sci* **26**, 1574-1583 (2017).
